# Semiparametric Outcome Regression-Based Estimator of Mann-Whitney-type Causal Effect

**DOI:** 10.21203/rs.3.rs-8340013/v1

**Published:** 2026-02-02

**Authors:** Safiya S. Sani, Bryan S. Blette, Chun Li, Abubakar Yahaya, Hussaini G. Dikko, Abubakar Usman, Usman J. Wudil, Faisal Dankishiya, Nafi’u Hussaini, C. William Wester, Muktar H. Aliyu, Bryan E. Shepherd

**Affiliations:** 1 Department of Agronomy, Ahmadu Bello University (ABU), Zaria, Kaduna, Nigeria.; 2 Department of Biostatistics, Vanderbilt University Medical Center (VUMC), Nashville, TN, USA.; 3 Department of Population and Public Health Sciences, University of Southern California, Los Angeles, California, USA.; 4 Department of Statistics, Ahmadu Bello University (ABU), Zaria, Kaduna, Nigeria.; 5 Vanderbilt Institute for Global Health (VIGH), 2525 West End Avenue, Suite 750, Nashville, TN 37203-1738, USA.; 6 Department of Medicine, Aminu Kano Teaching Hospital (AKTH), Kano, Nigeria.; 7 Department of Mathematics, Bayero University Kano (BUK), Kano, Nigeria.; 8 Department of Medicine, Division of Infectious Diseases, Vanderbilt University Medical Center (VUMC), Nashville, TN 37203-1738, USA.; 9 Department of Health Policy, Vanderbilt University Medical Center (VUMC), Nashville, TN, USA.

**Keywords:** Albuminuria, Causal Effect Estimation, Cumulative Probability Model, Estimated Glomerular Filtration Rate, Mann-Whitney-Type Parameter, Semiparametric Models, Urine Albumin to Creatinine Ratio

## Abstract

We introduce a novel semiparametric estimator for Mann-Whitney-type causal effects based on the cumulative probability model (CPM). CPMs are rank-based, invariant to monotone transformations of the outcome, and offer flexible outcome regression under confounding. We formalize the estimation under causal consistency, no interference, ignorability, and positivity, and develop accompanying inference procedures. Through simulations with varying sample sizes and effect magnitudes, the CPM estimator shows reduced variability and improved predictive accuracy relative to mis-specified parametric transformations. We demonstrate its applicability in a large cohort of People with HIV (PWH) in Northern Nigeria by assessing the causal effect of HIV status on albuminuria levels. Overall, our results highlight the value of robust semiparametric methods for causal inference in observational settings beyond average treatment effects. Findings should be interpreted in light of the observational design and the potential for unmeasured confounding.

## 1 Introduction

The Neyman-Rubin Causal Model (Rubin, 1974; Imbens and Rubin, 2015), also known as the potential outcomes framework, is a fundamental approach to define, identify, and estimate causal effects. For each subject i in a sample with a binary treatment, there are two potential outcomes, $Y_{i}(1)$ being the outcome if the subject is treated and $Y_{i}(0)$ the outcome if the subject is not treated. These are called potential outcomes because they represent what could potentially happen under each treatment condition. When treatment is applied to a subject, we observe $Y_{i}(1);Y_{i}(0)$ becomes the counterfactual outcome, representing what would have happened if the subject had not been treated. Conversely, if the subject is not treated, we observe $Y_{i}(0)$ and $Y_{i}(1)$ becomes counterfactual. The individual causal effect (ICE) can be defined as $Y_{i}(1)-Y_{i}(0)$, but each subject can only belong to one group at a time. Thus, identification of ICEs is not possible without very strong assumptions since we can never observe both $\left\{Y_{i}(1),Y_{i}(0)\right\}$ simultaneously.

Since ICEs cannot be directly observed, researchers often focus on estimating population-level causal effects. These are calculated by summarizing the individual-level causal effects across the entire population or a subset of the population. Some examples from Grilli and Rampichini (2010) include the Average Causal Effect (ACE): the expected difference in outcomes between treated and untreated subjects in the population, given by E[Y(1)−Y(0)] and the difference between the medians of the potential outcomes under the two treatment conditions, i.e., (median(Y(1))−median(Y(0))).

These estimands are termed “causal” because they were derived from comparisons between potential outcomes corresponding to different causal scenarios (i.e., outcomes under treatment versus no treatment). Such estimands enable quantifying the average or typical effect of a treatment within a population, even though individual causal effects cannot be directly observed. This framework underpins a variety of methods in causal inference, including those applied in both randomized experiments and observational studies.

While causal inference methods traditionally focus on estimating ACEs, there is growing interest in exploring alternative effect measures. The Mann-Whitney U test, also known as the Wilcoxon rank sum test, is a nonparametric statistical test that compares two independent samples. The Mann-Whitney U test is applicable to any outcome variable with a natural ordering, but it is particularly useful for comparing data outcomes that are skewed or ordinal. Beyond significance testing, the Mann-Whitney U test yields an interpretable parameter, the probabilistic index, which is the probability that a randomly selected subject in one group has a higher outcome value than a randomly selected counterpart in the other group. A probabilistic index greater than 0.5 suggests that treated units tend to have higher outcomes than controls. If the value is close to 0.5, there is little evidence of existence of any difference between the groups (i.e., similar outcomes between treated and control units). If it is significantly below 0.5, it indicates that controls tend to have higher outcomes.

The Mann-Whitney U test does not directly test medians but compares entire distributions and is valid even with tied data, contrary to common misconceptions (Divine *et al*. (2018)). Thas *et al*. (2012) introduced a semiparametric model of the probabilistic index, the probabilistic index model (PIM), which models the probability that the outcome Y given one set of covariates is less than an independent outcome $Y^{\prime }$ given another set of covariates. Vermeulen *et al*. (2015) discussed enhancing the power of Mann-Whitney U test in randomized experiments, particularly for skewed outcomes or for small sample sizes, and proposed a method to adjust for auxiliary baseline covariate information, which are often collected but not used in the traditional Mann-Whitney U test. While these advances promoted the use of auxiliary covariates, they did not formally describe a relevant causal inference framework that could be used in observational study settings.

In contrast, Fay *et al*. (2018) and Zhang *et al*. (2019) proposed a Mann-Whitney-type causal effect estimand denoted by θ, and measured as $P\left\{Y_{i}(1)>Y_{j}(0)\right\}$, with subscripts i and j representing two independent subjects. Thus, θ captures the tendency of a randomly selected unit to have a higher potential outcome under treatment than a different randomly selected unit’s potential outcome under control. Zhang *et al*. (2019) proposed several methods for estimating the Mann-Whitney-type causal effect, one of which is an estimator based on an outcome regression (OR) model, which models the conditional distribution of the outcome (perhaps after a Box-Cox transformation) given treatment and confounders. The OR estimator of the Mann-Whitney-type causal effect proposed by Zhang *et al*. (2019) requires correct model specification for unbiased estimation and was presented using parametric models. For example, with skewed data, their approach requires choosing an appropriate transformation such that the transformed response data are normally distributed and then fitting a normal linear model to the transformed data. However, selecting a transformation and then fitting a parametric model is contrary to the spirit of the Mann-Whitney test, which is invariant to any monotonic transformation of the data and requires no distributional assumptions.

In this paper we explore the use of a semiparametric model that is invariant to outcome transformation, the cumulative probability model (CPM), for obtaining an OR estimator of the Mann-Whitney-type causal parameter, providing a robust and flexible approach to causal effect estimation. CPMs are often fit to ordinal outcomes (e.g., the proportional odds model is a CPM), but Liu *et al*. (2017) demonstrated that CPMs could also be used to analyze response variables that are continuous, or mixtures of ordinal and continuous (e.g., data with detection limits). Fitting a CPM to continuous data is equivalent to fitting a semiparametric linear transformation model, where data are assumed to follow a linear model after some unspecified monotonic transformation that is nonparametrically estimated. Liu *et al*. (2017) highlighted the robustness and flexibility of CPMs, which motivates our use of CPMs in causal effect estimation. Li *et* al. (2023) established asymptotic properties of CPMs for continuous outcomes. Here we explore a novel approach to estimating the Mann-Whitney-type causal effect measure using the CPM. We develop an innovative estimator that leverages CPM-based regression models for causal effect estimation.

The remainder of the manuscript is organized as follows. Section 2 describes the CPM and the proposed estimator of the Mann-Whitney-type causal effect for observational studies. In section 3, we present simulation experiments to evaluate the performance of this estimator and to compare it with other estimators. Section 4 presents an application of the method to an observational study, estimating the causal effect of HIV on kidney function among adult People Living with HIV (PWH) seen at the Aminu Kano Teaching Hospital (AKTH) in Northern Nigeria. The paper concludes with section 5 wherein additional discussion of the CPM estimator of the Mann-Whitney-type effect measure is provided.

## 2 Methodology

### 2.1 Mann-Whitney-type Causal Effect Estimand

We examine a scenario with two groups: the exposed, denoted as A = 1, and the unexposed, denoted as A = 0, with an orderable (i.e., continuous, ordinal, or a mixture of the two) outcome represented by Y. We define $Y_{i}(1)$ as the potential outcome for individual i if assigned to the treatment group (A = 1) and $Y_{i}(0)$ as the potential outcome for individual i if assigned to the control group (A = 0). Additionally, let X be a vector of covariates that are unaffected by treatment. Our focus is on estimating a population level Mann-Whitney-type causal effect.

Let $F_{Y(0)}(y)$ and $F_{Y(1)}(y)$ be the marginal distributions of Y(0) and Y(1), respectively. The Mann-Whitney-type effect estimand is represented in terms of the marginal distributions of potential outcomes as:

(1) $$\theta =E\left\{\text{h}\left(Y_{i}(1),Y_{j}(0)\right)\right\}=\iint \text{h}\left(y_{1},y_{0}\right)dF_{Y(1)}\left(y_{1}\right)dF_{Y(0)}\left(y_{0}\right)$$

where

(2) $$\text{h}\left(y_{1},y_{0}\right)=I\left(y_{1}>y_{0}\right)+0.5I\left(y_{1}=y_{0}\right)$$

and I(.) is an indicator function. For a continuous outcome, $\text{h}\left(y_{1},y_{0}\right)=I\left(y_{1}>y_{0}\right)$ and the corresponding θ, namely, $P\left(Y_{i}(1)>Y_{j}(0)\right)$ captures the tendency of a randomly selected unit, subject i, to have a higher potential outcome under treatment than the potential outcome under control of an independent randomly selected unit, subject j. Note that θ depends only on marginal distributions of the potential outcomes because subscripts i and j represent two independent subjects.

Since θ is defined using the marginal distributions of the potential outcomes, to identify this estimand, we first identify $F_{Y(a)}(y)$ with observable data. To do this, we make the following four common causal assumptions:

1. **No interference:** The treatment received by one individual does not affect the outcomes of another individual.
2. **Consistency:** The potential outcome for an individual under a specific treatment condition matches the observed outcome when that individual receives that treatment:
Y=Y(a)ifA=a.
3. **Ignorability:** Treatment assignment, A, is independent of the potential outcomes of Y conditional on the covariates vector X (Rosenbaum & Rubin, 1983). This is an assumption of no unmeasured confounding. This can be expressed as:
(Y(1),Y(0))⫫A∣X
4. **Positivity:** Given X, there is a non-zero probability of being assigned the treatment or control:
0<P(A=1∣X)<1

Under these assumptions, the marginal distribution of Y(a), a=0 or 1 is

(3) $$F_{Y(a)}(y)=P(Y(a)\leq y)=\int P(Y(a)\leq y|X=x)dF_{X}(x)\;F_{Y(a)}(y)=\int P(Y(a)\leq y|A=a,X=x)dF_{X}(x)\;=\int P(Y\leq y|A=a,X=x)dF_{X}(x)$$

The second equality is from the law of total probability (conditioning on X), the third line is from Ignorability, and the fourth is from Consistency. Thus, the marginal distribution of Y(a) is now written in terms of observables (not counterfactual variables), so it is identified and can be estimated.

The distribution of $X,F_{X}(x)$ can be estimated without modeling assumptions using the empirical cumulative distribution function of X. However, with continuous or multi-dimensional X, modeling assumptions will typically be needed to estimate P(Y≤y∣A=a,X=x). Ideally, to maintain the spirit of the rank-based nature of θ, the model for P(Y≤y∣A=a,X=x) will also be based on ranks. In the next section, we describe such a model.

### 2.2 Semiparametric Cumulative Probability Model

We assume that the outcome Y follows a semiparametric linear transformation model, that is, $Y=H\left(\beta^{T}X+\tau A+\varepsilon \right)$. H is a transformation function that is unknown but assumed to be monotonic increasing, β is a vector of coefficients, τ is the coefficient associated with the treatment variable and ε is a random variable that is assumed to follow a cumulative distribution function $F_{\varepsilon }$ and is independent of X and A. For ease of presentation, we have chosen to write this model with simple linear relationships, but we should note that the model could easily be made more flexible by including, for example, pre-specified functions of X (e.g., polynomials, splines, products) and interactions between functions of X and A. It follows that $H^{-1}(Y)=\beta^{T}X+\tau A+\varepsilon$, where $H^{-1}(Y)$ is a transformation of Y such that the transformed value is linearly related to X and A. We specify the cumulative probability distribution of Y conditional on X and A as:

(4) $$F(y|X,A)=P(Y\leq y|X,A)=P\left[H\left(\beta^{T}X+\tau A+\varepsilon \right)\leq y|X,A\right]\;=P\left[\varepsilon \leq H^{-1}(y)-\beta^{T}X-\tau A|X,A\right]=F_{\varepsilon }\left(H^{-1}(y)-\beta^{T}X-\tau A\right)$$

By letting $H^{-1}(y)=\alpha (y)$ and $G(.)={{\text{F}}_{\varepsilon }}^{-1}(.)$, we can write the linear transformation model as a CPM:

(5) $$G[P(Y\leq y|X,A)]=\alpha (y)-\beta^{T}X-\tau A,$$

where α(y) is sometimes referred to as an intercept function and G(.) is a link function. We fit our CPM in a semiparametric manner that uses a step function to estimate α(y). Without loss of generality, for n ordered unique observed values of Y denoted by $y_{1}<y_{2}<\ldots <y_{\text{n}}$, we estimate n associated intercepts, $\alpha_{1}<\alpha_{2}<\ldots <\alpha_{\text{n}-1}<\alpha_{\text{n}}$, where $\alpha_{\text{i}}=\alpha \left({\text{y}}_{\text{i}}\right),\text{i}=1,\cdots ,n$. We express the CPM as:

(6) $$G[P(Y\leq y|X,A)]=\alpha_{i}-\beta^{T}X-\tau A.$$

The nonparametric likelihood function of the CPM for independent and identically distributed realizations of ${(X}_{i},\;{\text{A}}_{i},{\text{Y}}_{i})$ is given by:

(7) $$L(\beta ,\tau ,\alpha )=\prod_{i=1}^{n}\left\{G^{-1}\left(\alpha_{i}-\beta^{T}X_{i}-\tau A_{i}\right)-G^{-1}\left(\alpha_{i-1}-\beta^{T}X_{i}-\tau A_{i}\right)\right\}$$

In this equation, $\alpha_{0}$ is an auxiliary parameter with the constraint $\alpha_{0}<\alpha_{1}$. The likelihood function reaches its maximum when $\alpha_{0}=-\infty$ and $\alpha_{n}=\infty$ allowing us to simplify it to:

(8) $$L(\beta ,\tau ,\alpha )=G^{-1}\left(\alpha_{1}-\beta^{T}X_{1}-\tau A_{1}\right)\times \overset{n-1}{\underset{i=2}{\prod }}\left[G^{-1}\left(\alpha_{i}-\beta^{T}X_{i}-\tau A_{i}\right)-\right.\left.G^{-1}\left(\alpha_{i-1}-\beta^{T}X_{I}-\tau A_{i}\right)\right]\times \left[1-G^{-1}\left(\alpha_{n-1}-\beta^{T}X_{n}-\tau A_{n}\right)\right].$$

This formulation is analogous to the multinomial likelihood for cumulative link models when outcomes are considered as ordered categorical. Maximizing the function results in the nonparametric maximum likelihood estimators (NPMLEs) for $\alpha =\left(\alpha_{1},\alpha_{2},\ldots ,\alpha_{\text{n}-1}\right),\beta$, and τ. The NPMLE of the transformation function α(y) forms an increasing step function with a step corresponding to each of the n−1 intervals between adjacent outcome values from $y_{1}$ to $y_{n}$. The model applies to every distinct outcome value, with each value associated with a corresponding α. Because the transformation is nonparametrically estimated, the estimated parameters τ and β are invariant to any monotonic transformation of Y. Thus, the CPM is rank-based. With a single binary predictor X, the score test for the corresponding β in the CPM is nearly identical to the Mann-Whitney/Wilcoxon rank sum test (McCullagh 1980, Tian et al., 2024). With a bounded range of Y, the maximum likelihood estimators of α,β, and τ are consistent and asymptotically normally distributed (Li et al., 2023). Estimation can be done using the ˋormˋ function in the ˋrmsˋ package (Harrell, 2025) in R software (R Core Team, 2025).

### 2.3 Estimating Mann-Whitney-like causal effects using CPMs

Estimating the distributions of potential outcomes, $F_{Y(a)}(y)$, follows the maximum likelihood estimation of the model parameters. We do that by replacing the parameters in the RHS of (6) by their respective estimators. Then $F_{Y(a)}(y)$ can be estimated using a plug-in estimator for (3) as:

(9) $${\hat{F}}_{Y(a)}(y)=\frac{1}{n}\sum_{i=1}^{n}F_{\varepsilon }\left(\overset{ˆ}{\alpha }(y)-{\overset{ˆ}{\beta }}^{T}x_{i}-\overset{ˆ}{\tau }a\right)$$

for a=0 or 1, n is the total sample size, and $\overset{ˆ}{\alpha }(y)$ is a step function with jumps at the observed y, specifically $\overset{ˆ}{\alpha }(y)=\overset{ˆ}{\alpha }\left(y_{i}\right)={\overset{ˆ}{\alpha }}_{i}$, where $y_{i}=\text{max}\left\{y_{1},\ldots ,y_{n}\right\}$ such that $y_{i}\leq y$.

To estimate our Mann-Whitney causal effect parameter, θ, we use plug-in estimators for (1) based on the estimated marginal CDFs of the potential outcomes. Specifically,

(10) $${\hat{\theta }}_{REG}^{CPM}=\sum_{i=1}^{n}\sum_{j=1}^{n}\;\text{h}\left(y_{i},y_{j}\right)\left[{\hat{F}}_{Y(1)}\left(y_{i}\right)-{\hat{F}}_{Y(1)}\left(y_{i-1}\right)\right]\times \left[{\hat{F}}_{Y(0)}\left(y_{j}\right)-{\hat{F}}_{Y(0)}\left(y_{j-1}\right)\right],$$

where ${\hat{F}}_{Y(a)}\left(y_{0}\right)=0$ and ${\hat{F}}_{Y(a)}\left(y_{n}\right)=1$. An alternative formulation would exclude i=j from the double summation to avoid ties; with large n, this alternative is equivalent to equation (10), which we use throughout this manuscript. Equation (10) leverages the flexibility and robustness of CPMs to estimate the Mann-Whitney-type causal effect. The CPM-based regression estimator is particularly advantageous because it does not require the specification of a parametric model for the outcome distribution, making it invariant to outcome transformation and more robust to model misspecification. Under the same assumptions as Li *et al*. (2023), our CPM-based outcome regression estimator of θ is consistent and asymptotically normal. Confidence intervals can be constructed using the bootstrap.

## 3 Simulations

### 3.1 Simulation Procedure

We performed a simulation study to evaluate the performance of the proposed method. We estimated performance metrics based on 1000 simulated datasets for each of a variety of scenarios. Each dataset had a sample size of n = 50, 200 or 500. A single covariate, X, normally distributed and having zero mean and unit variance was generated. A binary treatment and a skewed continuous outcome were then generated. The binary treatment variable, A, was generated according to a logistic model of the form:

logit{P(A=1∣X)}=(1,X)γ

where $\gamma =(-0.5,0.75{)}^{T}$. The outcome, Y, was generated from a normal linear model exponentially transformed as:

$$Y=\text{exp}(\beta X+\tau A+\varepsilon ),\;\varepsilon N\left(0,1^{2}\right),$$

where β∈{0,0.5,2} and τ∈{0,0.5,2}; varying β and τ control the amount of confounding and the strength of the treatment effect. Note that this generates data following the linear transformation model outlined in Section 2.1, with H(.)=exp(.). Different values of β and τ lead to different values for the causal Mann-Whitney parameter, θ. When τ=0,θ=0.5, indicating that the treatment has no causal effect. For other values of τ, true θ values were determined empirically as outlined in the Web Appendix.

The causal Mann-Whitney parameter was estimated using the statistical methods described in Section 2. CPMs were fit using a properly specified probit link function (inverse of a standard Gaussian distribution, $\Phi^{-1}(y)$) without specifying the transformation H(.) (i.e., it was empirically estimated) using the ‘orm(.)’ function in the ˋrmsˋ package (Harrell, 2025) in R software (R Core Team, 2025).

Ninety-five percent confidence intervals for our estimates of θ were computed as the estimate ±1.96 times the standard deviation of 200 replicate bootstrap estimates. We considered the robustness of our approach by performing estimation fitting CPMs that incorrectly specified the link function (using logit link instead of probit link) and that failed to include the confounder variable, X (i.e., β incorrectly constrained to zero). We investigated the efficiency of our approach by comparing it to a properly specified parametric model (e.g., OR estimator of Zhang *et al*. (2019) after properly log-transforming the data and fitting a normal linear model). Because the proper transformation is unknown in practice, we also fit an incorrectly specified parametric model that applied the OR estimator of Zhang *et al*. (2019) by fitting a normal linear model after incorrectly square-root-transforming the outcome. For the specifications of these comparison models, see Web Appendix II.

For each simulation scenario, the standard deviation (SD), root mean squared error (RMSE), and bias of the estimates were computed. Coverage probabilities (CP) were also computed. The relative efficiency of the CPM estimator to both an estimator from a correctly and an incorrectly transformed parametric model was assessed using SD and RMSE ratios of the two estimators. All performance metrics were calculated with respect to the true value of θ. All analyses were conducted using R Software Version 4.4.1 (R Core Team, 2025) and interface RStudio Version 4.2.7 (RStudio Team, 2024) on a Windows 11 Pro platform (Microsoft Corporation, 2021).

### 3.2 Simulation Results

Simulation results are presented in the tables that follow. The β column represents the confounder coefficient in the model, τ is the corresponding treatment coefficient, θ is the theoretical value (true parameter) of the Mann-Whitney causal effect being estimated. Bias is calculated as the difference between the average of our estimated values and θ. SD indicates the standard deviation of the estimates of the parameter across the 1000 simulated datasets. The RMSE assesses the average magnitude of the errors between the estimated values and the true values. Lower RMSE values suggest better model performance. The coverage probability (CP) indicates the proportion of times that the estimated 95% confidence intervals contained the true parameter value θ. A CP value close to 0.95 signifies good performance.

Table 1 shows the performance of our method with properly specified CPMs across multiple sample sizes, levels of confounding, and effect sizes. In general, our procedure performed well across a variety of values of θ, even with sample sizes as small as 50. Bias was low at all sample sizes but tended to be lower at larger sample sizes. Coverage probability was close to the nominal 0.95 level, particularly at the larger sample sizes.

Table 2 presents the performance of our method using the improperly specified logit link function, rather than probit, for the cumulative probability models. Bias remained low and coverage probability was still close to the 0.95 mark, at all sample sizes and true values of θ. Overall, these results demonstrate the estimator’s robustness to misspecification of the link function.

Table 3 shows the performance of our method when employing improperly specified linear predictors, neglecting to include the confounder variable, for the cumulative probability models. As expected, bias was high and coverage low except in the scenario where the confounder effects were absent (i.e., β=0). These findings underscore the necessity of properly accounting for confounders to maintain estimator validity.

Table 4 compares the bias of estimators obtained from properly specified CPMs and those derived from correctly transformed parametric models, while also presenting their relative efficiencies. Bias was similarly low for the proposed estimator and that based on the correctly transformed outcome regression model. The SDs of the CPM-based estimates tended to be slightly larger than those based on the correctly transformed outcome regression models, but the SD ratios were less than 1 in a few scenarios. Same applies to the RMSE ratios. This suggests a minor loss in efficiency when employing the CPM approach compared to the correctly specified parametric model. However, the robustness of the CPM approach is noteworthy, as it does not require any data transformation. Table 5 compares the biases of estimators obtained from properly specified CPMs and those derived from incorrectly transformed parametric models, while also presenting their relative efficiencies. In these simulations, the CPM estimator has substantially lower bias than those based on the incorrectly transformed parametric model; it also has RMSE ratios well below 1 in most settings.

## 4 Application

In this section, we present an application of the method to an observational study of kidney disease *(Etiology of* *P**ersistent* *Micro**albuminuria in Nigeria (P-MICRO) Study; DK127912*), estimating the causal effect of HIV on urine albumin-to-creatinine ratio (uACR) and estimated glomerular filtration rate (eGFR) among study participants seen at AKTH in Northern Nigeria.

The P-MICRO study was initiated following an evaluation of the *Renal Risk Reduction (R3) Trial* (DK112271) findings in Nigeria. The R3 Trial found that persistent albuminuria was common among ART-treated PWH. Microalbuminuria is an independent risk factor for cardiovascular and kidney disease and a predictor of end-organ damage, both in the general population and among persons living with HIV (PWH) (Matsushita *et al*., 2010; Fox *et al*., 2012; Khosla, Sarafidis & Bakris, 2006; Mann, Yi & Gerstein, 2004). Defined as an albumin-to-creatinine ratio (uACR) of 30–300 mg/g, microalbuminuria can signify early glomerular damage or microvascular endothelial dysfunction and is used in the early detection of kidney disease (Baweja *et al*., 2011; Rodriguez *et al*., 1988). HIV is hypothesized to increase the risk of albuminuria, likely due to HIV-associated nephropathy and related comorbidities, as well as the use of antiretroviral therapy (ART), which—while universally prescribed to control the virus—may contribute to increased albuminuria as a side effect. Among PWH, albuminuria has been associated with increased systemic T-cell activation and more rapid progression to AIDS (Gerstein, Mann, Yi, *et al*., 2001; Choi *et al*., 2010; Scherzer *et al*.; Reins *et al*., 2014). Microalbuminuria is also an important risk factor for mortality in ART-treated PWH, likely as a marker of inflammation and endothelial activation (Keane & Eknoyan, 1999; Wyatt *et al*., 2010). A cohort of adult ART-treated PWH and a cohort of age- and sex-matched HIV-negative adults were recruited from AKTH (Wester *et al*., 2022). Our analysis uses cross-sectional (baseline) P-MICRO (DK127912) data from 2998 study participants, of whom 2,248 (75%) are PWH.

Our exposure variable is HIV status (negative/positive), and the outcome variables are uACR and eGFR, both of which are commonly used to detect albuminuria (uACR) and assess kidney function (eGFR). Typically, uACR values are right-skewed (Tsuchihashi *et al*., 2002, and Kim *et al*., 2023), making a Mann-Whitney-type effect estimate an appropriate choice (see Figure 1 in Web Appendix III). In contrast, eGFR values typically exhibit a left-skewed (negatively skewed) distribution in the general population (Takahashi *et al*., 2016, and Chu *et al*., 2021; see Figure 2 in Web Appendix III). In practice, uACR is often dichotomized as normoalbuminuria (<30 mg/g), microalbuminuria (30–300 mg/g), and macroalbuminuria (>300 mg/g). Although categorizing a continuous variable is generally discouraged, we demonstrate the flexibility of our method by also applying it to this ordered categorical variable.

We consider the following baseline covariates assumed to be sufficient to satisfy the conditional exchangeability assumption: age (in years), body mass index (BMI), ethnicity (Fulani, Hausa, Igbo, Yoruba, Other), sex (male/female), alcohol use (yes/no), smoking status (yes/no), hepatitis B infection (yes/no), Apolipoprotein-1 (*APOL1*) genetic risk allele/variant status (with these variants being found on chromosome 22) (categorized as high risk [HR] genotype for 2 copies of *APOL1* risk variants versus low-risk [LR] genotype for 0 or 1 copy of *APOL1* risk variants), hypertension (categorized according to the eighth edition of the Joint National Committee guidelines into four groups: normal, prehypertension, stage 1 hypertension, and stage 2 hypertension), diabetes mellitus (yes/no), concomitant medication use (other than HIV antiretroviral therapy) (yes/no), and other comorbid medical conditions (yes/no).

Tables 6a and 6b compare the performance of Mann-Whitney-type effect estimators using different modelling approaches (CPMs with various link functions and parametric models with different transformations) to evaluate the effect of HIV on uACR and eGFR respectively, adjusted for potential confounders. At a 95% confidence level, 50 bootstrap replications were used to compute the confidence intervals.

With uACR as a continuous outcome variable, CPM-based estimators provided very similar effect estimates across different link functions, with logit (CPM 1) and probit (CPM 2) models yielding nearly identical results. The log-log link function resulted in the best model fit (e.g., highest likelihood; see Supplementary Material). In contrast, parametric models showed variability, highlighting their sensitivity to transformation choice. Among them, NLM 2 produced estimates closest to CPMs, suggesting a log transformation may have been most appropriate. Overall, CPMs offer a robust and stable analysis approach, while parametric models require careful transformation selection to ensure reliable interpretations.

When uACR was categorized, the Mann-Whitney-type effect estimates remained very similar across link functions, closely resembling their continuous counterparts. This stability validates the reliability of CPM-based estimators in estimating causal effects, regardless of whether the outcome variable is treated as continuous or categorical. This finding supports the idea that CPMs provide a flexible framework for evaluating the effect of HIV on kidney function markers, without requiring parametric assumptions.

Our Mann-Whitney causal effect estimators based on the CPM suggest that HIV lowers eGFR (lower eGFR implies reduced kidney function.) This conclusion held, regardless of the link function used for the CPM. The estimated Mann-Whitney causal effect was slightly higher using the log-log link function. It should be noted that the logit link function resulted in the best model fit (e.g., highest likelihood; see Supplementary material). Interestingly, parametric models produced similar Mann-Whitney causal effect estimates to CPM-based estimators in this case. This suggests that the relationship between HIV and eGFR may be less dependent on transformation choices compared to uACR. The agreement between approaches reinforces the validity of the observed negative effect of HIV on kidney function.

## 5 Discussion

In this study, we proposed a semiparametric outcome regression-based estimator for the Mann-Whitney-type causal effect based on the CPM. We evaluated our estimator’s performance through simulations and with an application to an observational study of kidney disease. Our results demonstrate the robustness and reliability of the CPM-based estimator in different scenarios, highlighting its utility for observational studies.

Zhang *et al*. (2019) made a valuable contribution by proposing Mann-Whitney treatment effect estimators, providing researchers with a structured approach for estimating these causal effects. Zhang *et al*. (2019)’s OR estimator, which requires specifying a transformation and then assuming normality, highlights a critical consideration in causal inference: the trade-off between modeling assumptions and robustness. Parametric models offer simplicity and interpretability but can be restrictive and prone to model misspecification, especially with skewed data. The necessity to transform data to meet parametric assumptions can be cumbersome and may not always lead to accurate causal estimates. In contrast, semiparametric approaches such as our OR estimator based on the CPM are consistent with the spirit of the Mann-Whitney test, which does not require specific distributional forms or transformations, thereby providing robustness. This robustness is desirable for practical applications where the true distribution of the data is generally unknown and often difficult to model accurately, particularly when dealing with skewed data.

The simulation results showed that the CPM estimator performed effectively across various sample sizes and combinations of confounder strength and treatment effects, exhibiting decreased bias and standard deviation with larger sample sizes, which led to smaller root mean square error values. Coverage probabilities were consistently close to the nominal 0.95 level, reinforcing the reliability of pairing the estimator with the bootstrap for inference. Notably, the CPM estimator maintained low bias and high accuracy, even with strong confounders and treatment effects, making it valuable in studies with significant effects. However, occasional convergence failures were observed in some simulations when either the confounder or the treatment effects were large with link-function misspecification. Specifically, failures occurred in 0.5% of simulation replications when β=0.5 and τ=2; 2.4% when β=2 and τ=0.5; 1.6% when β=2 and τ=0; and 3% when β=2 and τ=2. Lack of convergence could serve as an indicator of link-function misspecification.

The CPM estimator demonstrated robustness to the misspecification of the link function; however, improper linear predictor specification resulted in poor performance. As with all causal models, care must be taken in selecting covariates and how to include them in regression models. CPMs offer a degree of flexibility over normal linear models because they do not require specifying a transformation of the outcome. However, like normal linear models, how the covariates are included in the models is important; CPMs have similar flexibility (e.g., splines, interaction, tree partitions) to normal linear models. We did not consider doubly robust estimators in this manuscript; in short, our outcome regression estimators based on CPMs could be made doubly robust following procedures presented by Zhang *et al*. (2019).

The application of the CPM estimator in the P-MICRO (DK127912) study offered valuable insights into the causal relationship between HIV status and albuminuria among ART-treated PWH in Northern Nigeria. The findings underscore the robustness of CPMs in estimating the effect of HIV on markers of kidney function. For uACR, CPMs produced stable and consistent results across modeling choices, avoiding the sensitivity to transformation assumptions observed with parametric models. The alignment of CPM-derived estimates in both continuous and categorized uACR outcomes further supports their reliability.

We found that uACRs were higher in ART-treated PWH compared to their HIV-negative counterparts. Relatedly, ART-treated PWH demonstrated lower eGFR, suggesting worse kidney function compared to HIV-negative individuals. The consistent association between HIV and increased uACR, and between HIV and reduced eGFR reinforces the importance of early renal function surveillance in HIV-positive populations. These findings provide evidence supporting the hypothesis that HIV increases the risk of albuminuria in PWH (National Kidney Foundation, 2025; Charles *et al*., 2018).

In conclusion, the semiparametric outcome regression-based estimator of the Mann-Whitney-type causal effect offers a robust and reliable framework for causal effect estimation in observational studies. Its performance across various scenarios and its successful application to the P-MICRO study underscores its potential for estimating causal effects in complex settings. This study contributes to the growing body of literature on causal inference methods by introducing a practical tool for researchers investigating causal relationships in observational data. Unlike parametric approaches, this tool does not require the correct transformation of the typically-unknown outcome transformation.

However, the proposed CPM-based Mann-Whitney-type estimator rests on standard causal identification assumptions—causal consistency, no interference, positivity, and ignorability. Although the method demonstrated robustness to certain misspecifications in simulations, convergence failures occurred in a minority of replications under strong confounding and link-function misspecification (e.g., up to 3% in some scenarios). Performance may be affected by unmeasured confounding, model misspecification of the covariate functional form, and limited sample sizes. Future research should address these issues and explore extensions to broader data-generating processes and outcomes. Additionally, future work could focus on improving computational efficiency and expanding applicability to other health outcomes and populations.

## Supplementary Files

This is a list of supplementary files associated with this preprint. Click to download.

- SupplementaryMaterialsSafiya.docx

## Figures and Tables

**Table 1:** Mann-Whitney-Type Causal Effects and Performance Metrics of Estimators from Correctly Specified CPMs

n	β	τ	θ	Bias	SD	RMSE	CP
50	0	0	0.500	−0.0106	0.0856	0.0859	0.941
	0.5	0	0.500	−0.0085	0.0795	0.0797	0.933
	2	0	0.500	−0.0091	0.0426	0.0436	0.954
	0	0.5	0.638	−0.0103	0.0839	0.0839	0.939
	0.5	0.5	0.624	−0.0064	0.0816	0.0816	0.932
	2	0.5	0.563	−0.0091	0.0429	0.0439	0.947
	0	2	0.921	−0.0160	0.0432	0.0461	0.962
	0.5	2	0.897	−0.0108	0.0486	0.0497	0.942
	2	2	0.737	−0.0067	0.0450	0.0454	0.955
200	0	0	0.500	−0.0010	0.0444	0.0444	0.942
	0.5	0	0.500	−0.0026	0.0385	0.0386	0.941
	2	0	0.500	−0.0031	0.0193	0.0196	0.953
	0	0.5	0.638	−0.0034	0.0412	0.0426	0.947
	0.5	0.5	0.624	0.0001	0.0378	0.0378	0.956
	2	0.5	0.563	−0.0024	0.0207	0.0208	0.944
	0	2	0.921	−0.0021	0.0195	0.0196	0.955
	0.5	2	0.897	−0.0011	0.0229	0.0229	0.948
	2	2	0.737	−0.0020	0.0223	0.0223	0.940
500	0	0	0.500	−0.0013	0.0286	0.0286	0.939
	0.5	0	0.500	−0.0008	0.0242	0.0242	0.947
	2	0	0.500	−0.0008	0.0125	0.0125	0.937
	0	0.5	0.638	−0.0117	0.026	0.026	0.940
	0.5	0.5	0.624	−0.0003	0.0244	0.0243	0.947
	2	0.5	0.563	−0.0010	0.0128	0.0128	0.940
	0	2	0.921	−0.0139	0.0123	0.0124	0.935
	0.5	2	0.897	−0.0014	0.0147	0.0148	0.946
	2	2	0.737	−0.0012	0.0133	0.0134	0.943

**Table 2.** Mann-Whitney-Type Causal Effects and Performance Metrics of Estimators from Link Function Mis specified CPMs.

n	β	τ	θ	Bias	SD	RMSE	CP
50	0	0	0.500	−0.0112	0.0853	0.0860	0.952
	0.5	0	0.500	−0.0070	0.0829	0.0831	0.940
	2	0	0.500	−0.0107	0.0435	0.0447	0.949
	0	0.5	0.638	−0.0116	0.0891	0.0899	0.932
	0.5	0.5	0.624	−0.0102	0.0833	0.0839	0.927
	2	0.5	0.563	−0.0077	0.0473	0.0479	0.942
	0	2	0.921	−0.0174	0.0430	0.0464	0.970
	0.5	2	0.897	−0.0129	0.0506	0.0522	0.940
	2	2	0.737	−0.0059	0.0487	0.0490	0.947
200	0	0	0.500	−0.0027	0.0436	0.1342	0.950
	0.5	0	0.500	−0.0023	0.0392	0.4014	0.947
	2	0	0.500	−0.0025	0.0201	0.0202	0.960
	0	0.5	0.638	−0.0034	0.0418	0.0420	0.952
	0.5	0.5	0.624	−0.0007	0.0385	0.0385	0.961
	2	0.5	0.563	−0.0009	0.0215	0.0215	0.948
	0	2	0.921	−0.0080	0.0205	0.0220	0.949
	0.5	2	0.897	−0.0054	0.0245	0.0251	0.946
	2	2	0.737	−0.0017	0.0226	0.0226	0.951
500	0	0	0.500	−0.0023	0.0292	0.0293	0.930
	0.5	0	0.500	−0.0022	0.0261	0.0262	0.937
	2	0	0.500	−0.0011	0.0126	0.0127	0.955
	0	0.5	0.638	−0.0030	0.0276	0.0278	0.935
	0.5	0.5	0.624	−0.0030	0.0258	0.0260	0.941
	2	0.5	0.563	−0.0020	0.0133	0.0135	0.938
	0	2	0.921	−0.0064	0.0128	0.0143	0.936
	0.5	2	0.897	−0.0047	0.0153	0.0160	0.949
	2	2	0.737	−0.0020	0.0138	0.0139	0.935

**Table 3.** Mann-Whitney-Type Causal Effects and Performance Metrics of Estimators from Linear Predictor Mis-specified CPMs.

n	β	τ	θ	Bias	SD	RMSE	CP
50	0	0	0.500	−0.0078	0.0812	0.0815	0.955
	0.5	0	0.500	0.0773	0.0822	0.1128	0.844
	2	0	0.500	0.1610	0.0746	0.1774	0.506
	0	0.5	0.638	−0.0180	0.0823	0.0843	0.937
	0.5	0.5	0.624	0.0670	0.0764	0.1103	0.821
	2	0.5	0.563	0.1560	0.0726	0.1721	0.447
	0	2	0.921	−0.0137	0.0427	0.0448	0.960
	0.5	2	0.897	0.0201	0.0393	0.0441	0.869
	2	2	0.737	0.1125	0.0550	0.1252	0.467
200	0	0	0.500	−0.0029	0.0398	0.0399	0.952
	0.5	0	0.500	0.0826	0.0400	0.0918	0.454
	2	0	0.500	0.1670	0.0382	0.1713	0.012
	0	0.5	0.638	−0.0006	0.0388	0.0387	0.952
	0.5	0.5	0.624	0.0757	0.0368	0.0841	0.454
	2	0.5	0.563	0.0861	0.0359	0.0933	0.347
	0	2	0.921	−0.0022	0.0194	0.0195	0.945
	0.5	2	0.897	0.0332	0.0172	0.0374	0.517
	2	2	0.737	0.1247	0.0263	0.1275	0.012
500	0	0	0.500	−0.0018	0.0262	0.0263	0.943
	0.5	0	0.500	0.0833	0.0268	0.0876	0.111
	2	0	0.500	0.1712	0.0247	0.1730	0.000
	0	0.5	0.638	−0.0011	0.0241	0.0241	0.950
	0.5	0.5	0.624	0.0784	0.0228	0.0817	0.091
	2	0.5	0.563	0.1648	0.0230	0.1664	0.000
	0	2	0.921	−0.0015	0.0124	0.0125	0.942
	0.5	2	0.897	0.0342	0.0110	0.0359	0.172
	2	2	0.737	0.1280	0.0165	0.1290	0.000

**Table 4.** Bias and Relative Efficiency of the CPM Estimator Compared to a Properly Transformed Parametric Model Estimator.

Bias						
n	β	τ	${\overset{ˆ}{\theta }}_{\text{REG}}^{\text{CPM}}$	${\overset{ˆ}{\theta }}_{\text{CORRECT}}^{\text{OR}}$	SD ratio	RMSE ratio
50	0	0	−0.013	−0.015	1.0006	0.9976
	0.5	0	−0.012	−0.014	1.0539	1.0480
	2	0	−0.007	−0.015	1.0955	1.0483
	0	0.5	−0.008	−0.021	1.0381	1.0093
	0.5	0.5	−0.006	−0.012	1.0033	0.9954
	2	0.5	−0.007	−0.014	1.0656	1.0252
	0	2	−0.016	−0.026	1.0007	0.9098
	0.5	2	−0.007	−0.019	1.0789	1.0292
	2	2	−0.006	−0.014	1.0725	1.0349
200	0	0	−0.002	−0.002	1.0242	1.0248
	0.5	0	−0.002	−0.002	1.0202	1.0206
	2	0	−0.003	−0.004	1.0419	1.0320
	0	0.5	−0.003	−0.004	1.0289	1.0577
	0.5	0.5	−0.002	−0.003	1.0330	1.0317
	2	0.5	−0.003	−0.003	1.0222	1.0184
	0	2	−0.004	−0.005	1.0543	1.0378
	0.5	2	−0.003	−0.004	1.0685	1.0565
	2	2	−0.002	−0.006	1.0315	1.0013
500	0	0	0.000	0.000	1.0214	1.0213
	0.5	0	−0.001	−0.001	0.9832	0.9838
	2	0	−0.002	−0.001	1.0289	1.0356
	0	0.5	0.000	−0.001	1.0208	1.0199
	0.5	0.5	−0.001	−0.001	1.0109	1.0111
	2	0.5	−0.001	−0.002	0.9718	0.9657
	0	2	−0.001	−0.002	1.0087	0.9968
	0.5	2	−0.001	−0.001	1.0544	1.0511
	2	2	−0.002	−0.001	1.0613	1.0631

**Table 5.** Bias and Relative Efficiency of the CPM Estimator to an Improperly Transformed Parametric Model Estimator.

Bias						
n	β	τ	${\overset{ˆ}{\theta }}_{\text{REG}}^{\text{CPM}}$	${\overset{ˆ}{\theta }}_{\text{INCORRECT}}^{\text{OR}}$	SD ratio	RMSE ratio
50	0	0	−0.013	−0.021	0.9839	0.9670
	0.5	0	−0.012	−0.026	0.9845	0.9504
	2	0	−0.007	−0.148	0.6784	0.2746
	0	0.5	−0.008	−0.039	0.9888	0.9052
	0.5	0.5	−0.006	−0.043	0.9385	0.8359
	2	0.5	−0.007	−0.185	0.6001	0.2297
	0	2	−0.016	−0.141	0.5261	0.2766
	0.5	2	−0.007	−0.173	0.5057	0.2463
	2	2	−0.006	−0.326	0.5109	0.1435
200	0	0	−0.002	−0.011	1.0220	0.9916
	0.5	0	−0.002	−0.020	0.9791	0.8736
	2	0	−0.003	−0.169	0.4994	0.1184
	0	0.5	−0.003	−0.029	0.9966	0.8517
	0.5	0.5	−0.002	−0.038	0.9509	0.7037
	2	0.5	−0.003	−0.214	0.4224	0.0947
	0	2	−0.004	−0.142	0.4210	0.1419
	0.5	2	−0.003	−0.183	0.4224	0.1273
	2	2	−0.002	−0.377	0.3799	0.0580
500	0	0	0.000	−0.009	1.0397	0.9807
	0.5	0	−0.001	−0.016	0.9619	0.8155
	2	0	−0.002	−0.175	0.3967	0.0710
	0	0.5	0.000	−0.026	0.9961	0.7086
	0.5	0.5	−0.001	−0.036	0.9468	0.5390
	2	0.5	−0.001	−0.226	0.3328	0.0547
	0	2	−0.001	−0.137	0.3882	0.0894
	0.5	2	−0.001	−0.184	0.3887	0.0806
	2	2	−0.002	−0.393	0.2948	0.0339

**Table 6a.** Comparison of Mann-Whitney-type Effect Estimates from Different CPMs and Parametric Models for Assessing the Impact of HIV on uACR

		Outcome = uACR continuous				Outcome = uACR categorised			
Model	Details	Estimate	SE	95% CI (Lower)	95% CI (Upper)	Estimate	SE	95% CI (Lower)	95% CI (Upper)
CPM 1	Logit link function	0.544	0.014	0.516	0.572	0.544	0.007	0.530	0.557
CPM 2	Probit link function	0.544	0.014	0.516	0.571	0.545	0.007	0.533	0.558
CPM 3	Loglog link function	0.588	0.012	0.564	0.612	0.547	0.006	0.535	0.560
NLM 1	No transformation	0.178	0.004	0.170	0.186	-	-	-	-
NLM2	Log-transformation	0.574	0.012	0.551	0.596	-	-	-	-
NLM 3	Square-root-transformation	0.344	0.010	0.324	0.365	-	-	-	-

Key to Model Types and Transformations

CPM = cumulative probability model; NLM = normal linear model assumed after transformation.

**Table 6b.** Comparison of Mann-Whitney-type Effect Estimates from Different CPMs and Parametric Models for Assessing the Impact of HIV on eGFR

Outcome = eGFR continuous					
Model	Details	Estimate	SE	95% CI (Lower)	95% CI (Upper)
CPM 1	Logit link function	0.340	0.013	0.315	0.366
CPM 2	Probit link function	0.346	0.013	0.320	0.372
CPM 3	Log-log link function	0.408	0.011	0.386	0.430
NLM 1	No transformation	0.343	0.009	0.325	0.361
NLM2	Log-transformation	0.358	0.010	0.338	0.378
NLM 3	Square-root-transformation	0.347	0.013	0.322	0.372

Key to Model Types and Transformations

CPM = cumulative probability model; NLM = normal linear model assumed after transformation.

## Data Availability

Simulation and data analysis code are publicly available at https://github.com/shepheb1/ArchivedAnalyses. Because of privacy concerns and data sharing laws/agreements, we cannot freely share the dataset used for the kidney disease study; these data can be made available upon request by contacting the study authors and completing necessary data use agreements. We adhere to the journal’s ethical and archiving policies.
